# Classification of Intervertebral Disc Disease

**DOI:** 10.3389/fvets.2020.579025

**Published:** 2020-10-06

**Authors:** Joe Fenn, Natasha J. Olby, The Canine Spinal Cord Injury Consortium (CANSORT-SCI), Sarah A. Moore

**Affiliations:** Author Affiliations: DACVIM-Neurology, Associate Professor, Neurology and Neurosurgery, Department of Veterinary Clinical Sciences, The Ohio State University College of Veterinary Medicine, Columbus OH, United States; DACVIM Neurology, Professor of Neurology/Neurosurgery; Distinguished Chair of Gerontology, Department of Clinical Sciences, North Carolina State University College of Veterinary Medicine, Raleigh, NC, United States; DACVIM-Neurology, Professor; Chair, and Head, Department of Small Animal Clinical Sciences, College of Veterinary Medicine and Biomedical Sciences, Texas A&M University, College Station, TX, United States; DAVCIM (Neurology), Assistant Professor of Neurology, Department of Veterinary Clinical Sciences, Purdue University College of Veterinary Medicine, West Lafayette, IN, Unites States; Professor Neurology & Neurosurgery; Professor in Small Animal Clinical Sciences, College of Veterinary Medicine, Texas A&M University, College Station, TX, United States; ACVIM—Neurology, Professor and Service Head, Neurology and Neurosurgery, Department of Veterinary Clinical Sciences, College of Veterinary Medicine, The Ohio State University, Columbus, OH, Unites States; Department of Clinical Sciences, Colorado State University, Fort Collins, CO, United States; Department of Clinical Science and Services, Royal Veterinary College, Hatfield, United Kingdom; The Royal Veterinary College, University of London, Hatfield, United Kingdom & CVS referrals, Bristol Veterinary Specialists at Highcroft, Bristol, United Kingdom; Institute of Veterinary Pathology, Faculty of Veterinary Medicine, Leipzig University, Leipzig, Germany; Division of Clinical Neurology, Department for Clinical Veterinary Medicine, Vetsuisse Faculty, University of Bern, Bern, Switzerland; Department of Small Animal Medicine and Surgery, University of Veterinary Medicine Hannover, Hannover, Germany/Europe; Neurology and Neurosurgery, Department of Veterinary Medicine and Surgery, University of Missouri, Columbia, MO, United States; Department of Small Animal Medicine and Surgery, University of Veterinary Medicine Hannover, Hannover, Germany/Europe; ^1^Department of Clinical Science and Services, Royal Veterinary College, London, United Kingdom; ^2^Department of Clinical Sciences, College of Veterinary Medicine, North Carolina State University, Raleigh, NC, United States

**Keywords:** IVD, chondrodystrophy, degeneration, Hansen, extrusion, protrusion

## Abstract

Intervertebral disc disease (IVDD) has been recognized in dogs since the 1800s, when the first descriptions of extruded disc material within the vertebral canal were published. In the intervening time our understanding of intervertebral disc pathology in dogs and cats has increased dramatically, with many variations of IVDD described. Whilst the volume of literature and collective understanding of IVDD has expanded, there has also been scope for confusion as the definition of intervertebral disc disease, with its myriad different manifestations, becomes more complicated. A large volume of literature has aimed to combine the use of histopathology, diagnostic imaging and clinical findings to better understand the various ways in which IVDD can be classified. Much of this research has focused on the classification of mechanisms of intervertebral disc degeneration, centering around the differences between, and overlaps in, IVDD in chondrodystrophic and non-chondrodystrophic dog breeds. However, with the increasing availability of advanced imaging modalities allowing more accurate antemortem diagnosis, the concept of IVDD has expanded to include other clinical presentations that may not fit into traditional models of classification of IVDD. This review aims to provide an up to date overview of both historical and current systems of IVDD classification, highlighting the important findings and controversies underpinning them.

## Introduction

Intervertebral disc disease (IVDD) is a broad term that is widely used in veterinary medicine and encompasses a range of lesions affecting the intervertebral disc (Table 1). Since the first descriptions of IVDD in the dog by Dexler in the late 1800s (1, 2), advances in understanding of the underlying etiology have resulted in a gradual evolution in terminology and systems of classification. Initial reports described the presence of cartilaginous material within the epidural space of the vertebral canal (so-called *enchondrosis intervertebralis*), which was subsequently found to be associated with degenerated nucleus pulposus (1, 3). Building on these findings in the 1940s and 50s, Hansen and Olsson made huge advances in understanding the nature of canine IVDD, proposing a system of classification based on histopathological degenerative changes that persists today (2, 4–7). They described two distinct types of IVD degeneration, namely chondroid and fibroid metaplasia, associated with specific breed and signalment signatures. This introduced the classification of dog breeds according to the type of IVDD that was more prevalent, into chondrodystrophic (chondroid metaplasia) and non-chondrodystrophic (fibroid metaplasia) breeds (5, 8). These insights led to the subsequent classification of IVDD into Hansen Type I and Hansen Type II herniations, a system that is still widely used in veterinary medicine (4, 5, 9). At the time of this initial work, Hansen and others used various terms to describe the subsequent displacement of IVD material into the vertebral canal, with “protrusion,” “extrusion,” “prolapse” and “herniation” often used interchangeably (2, 5). Indeed, Olsson stated as much in 1951, reporting that “Disc protrusion [is] a synonym for disc herniation and disc prolapse” (2). Since then, the term “IVD extrusion” has become more widely associated with the acute extrusion of nucleus pulposus from an IVD that has features of chondroid metaplasia, whilst “IVD protrusion” has largely been reserved chronic annulus fibrosus thickening originally associated with fibroid metaplasia. In contrast, the term “IVD herniation” continues to be largely used as an umbrella term without specificity to a particular type of degenerative change. For the purposes of this report, in consistency with recent veterinary and human literature (10, 11), we have therefore used IVD herniation as a non-specific term to encompass any type of localized IVD displacement. This basic terminology is outlined in Table 1.

**Table 1:** Glossary of words commonly used in the description of intervertebral disc disease and suggested definitions.

**Term used**	**Proposed definition**
Intervertebral disc (IVD) disease	Very broad, non-specific term suggesting clinical IVD herniation, subclinical IVD herniation, or IVD degeneration without herniation
IVD herniation/prolapse/displacement	Non-specific descriptors that can be used to describe any form of IVDD that results in loss of structural integrity with part of the IVD displaced beyond its normal boundaries—typically into the vertebral canal
IVD extrusion	Used to describe the herniation/prolapse/displacement of internal contents (predominantly nucleus pulposus) through the annulus fibrosus. Can be associated with varying degrees of degenerative change, hydration, or trauma
IVD protrusion	Used to describe the prolapse/herniation/displacement of the annulus fibrosus beyond its normal boundaries Typically associated with fibroid metaplasia (often referred to as Hansen Type II IVD herniation)

In recent years further advances in the understanding of IVDD have been made, building on the seminal early work of Hansen and Olsson. For example, the use of detailed histological grading systems has suggested that both chondrodystrophic and non-chondrodystrophic breeds of dog undergo chondroid metaplasia, albeit at very different rates and times (9, 12–14). Our understanding of the basis of early chondroid metaplasia in chondrodystrophic breeds has also improved dramatically with discovery of expression of a fibroblast growth factor 4 (*FGF*4) retrogene on chromosome 12 as a strong risk factor for IVD extrusion (15–17). In addition to these advances in histological and genetic descriptions of IVDD, advances in diagnostic techniques such as magnetic resonance imaging (MRI) have contributed to the expansion of the group of conditions that might reasonably be referred to as types of IVDD in dogs and cats. The improved diagnostic ability provided by these developments has led to the increasingly frequent diagnosis of IVD conditions that occur with minimal evidence of traditional features of disc degeneration, including the herniation of relatively well-hydrated nucleus pulposus material (18–20). This has created a degree of confusion, as numerous reports of similar clinical presentations have appeared in the literature under a variety of terminology (Table 2). This is perhaps best exemplified by the condition variably termed Hansen Type III IVD herniation, traumatic IVD extrusion and high-velocity low-volume IVD extrusion among others, before recently becoming more uniformly accepted as acute non-compressive nucleus pulposus extrusion (ANNPE) (18, 74–76). Whilst these terms are all used to indicate a peracute non-compressive extrusion of nucleus pulposus, traumatic IVD extrusion has also been applied to cases of compressive extrusion of degenerative nucleus pulposus following vertebral column trauma (77). Alongside ANNPE, further conditions that can be considered as types of IVDD have also appeared with increasing frequency in the literature, such as intradural or intramedullary disc extrusions (IIVDE) and acute compressive hydrated nucleus pulposus extrusions (HNPE) (20, 88, 101). In this article we have utilized updated and consistent terminology for these conditions that builds on the traditional two-type IVDD classification model.

**Table 2:** Terminology used in the veterinary literature to describe different types of intervertebral disc (IVD) disease.

**Proposed/current consensus**	**Examples of reported terms used**	**References**
(Hansen Type I/Acute) IVD extrusion	Hansen Type I IVD herniation	(9, 21–24)
	Hansen Type I IVD extrusion	(25–31)
	Hansen Type I IVD disease	(32–36)
	(Acute-) IVD herniation	(24, 37–46)
	(Acute-) IVD extrusion	(33, 34, 41, 47–53)
	(Acute-) IVD disease	(54–58)
	IVD protrusion	(54, 59, 60)
	Type I protrusion/prolapse	(5)
(Hansen Type II/Chronic) IVD protrusion	Hansen Type II IVD herniation	(9, 22, 23, 61)
	Hansen Type II IVD protrusion	(29, 61, 62)
	Hansen Type II IVD disease	(62)
	IVD protrusion	(23, 48, 51, 61, 63–66)
	Type II protrusion/prolapse	(5)
Acute IVD extrusion (Hansen Type I) with extensive epidural hemorrhage	Acute IVDE with extensive epidural hemorrhage	(37, 67)
	Disc extrusion with extensive epidural hemorrhage (DEEH)	(67)
	Epidural spinal hematoma and IVD extrusion	(68)
Acute non-compressive nucleus pulposus extrusion (ANNPE)	Acute non-compressive nucleus pulposus extrusion (ANNPE)	(18, 35, 52, 69–73)
	Hansen Type III IVDD	(74)
	High-velocity, low-volume IVD extrusion	(75)
	Traumatic IVD extrusion	(76, 77)
	IVD explosion	(78)
	Traumatic IVD prolapse	(5)
	Missile disks	(79, 80)
Hydrated nucleus pulposus extrusion (HNPE)	Hydrated nucleus pulposus extrusion (HNPE)	(19, 20, 81, 82)
	Hydrated nucleus pulposus herniation	(83)
	Acute compressive hydrated nucleus pulposus extrusion	(84)
	Partially degenerated disc extrusion	(85)
	Intraspinal cyst	(86, 87)
	Canine discal cyst	(86)
Intradural/intramedullary IVD extrusion (IIVDE)	Intradural/intramedullary IVD extrusion (IIVDE)	(88)
	Intradural IVD herniation	(72, 89, 90)
	Intramedullary IVD herniation	(91)
	Intramedullary IVD extrusion	(92, 93)
Traumatic IVD extrusion	Traumatic IVD extrusion	(76, 77)
	Traumatic IVD prolapse	(5)
Fibrocartilaginous embolic myelopathy (FCEM)	Fibrocartilaginous embolic myelopathy (FCEM)	(79, 88, 94, 95)
	Fibrocartilaginous embolism (FCE)	(96–99)
	Ischaemic myelopathy	(69, 77, 96, 100–102)
	Spinal cord infarction	(103)

As genetic investigations continue and advanced imaging techniques improve, it is likely that this terminology will evolve further. As a result, it is important to periodically review recent and historical literature surrounding IVDD classification with the aim of developing intuitive terminology that reduces confusion and allows a better understanding of the underlying etiology. Specifically, a better understanding of disease classification and terminology is vital in order to facilitate effective research efforts, allowing optimal study design and case selection for clinical, molecular and histological investigations aimed at ultimately improving patient welfare and treatment outcomes.

Whilst there are still many controversies in the categorization of types of disc-associated lesions in veterinary medicine, in this article we aim to review the relevant literature and to outline a consensus where possible. We will provide an up to date overview of reported types of IVDD, highlighting important features, and aiming to explain the theories behind their classification.

## Anatomy of the Intervertebral Disc

The intervertebral disc plays a critical role in stability of the vertebral column, effectively binding individual vertebrae together to provide support for the entire axial skeleton while allowing multiplanar movement. Additionally, it protects the spinal cord and facilitates the exit/entrance of peripheral nerves. Embryologically it derives from the mesoderm with the exception of the nucleus pulposus, which is a remnant of the notochord (104, 105). The intervertebral disc has 4 regions, all of which contribute to its complex role; the nucleus pulposus, the transitional zone, the annulus fibrosus and the cartilaginous endplates (22).

### The Nucleus Pulposus

The nucleus pulposus is a gelatinous, bean shaped mass that sits slightly dorsally within the intervertebral disc (Supplementary Figure 1). While bounded on all sides by the transitional zone, cranially and caudally it lies close to the cartilaginous endplates. It is derived from the notochord and is characterized by an extremely high-water content, up to 88% in the young animal. Islands of physaliferous notochordal cells can be seen within the ground substance in the young animal and are gradually replaced by chondrocyte like cells with age (5, 22, 106). Notochordal cells produce low levels of type 2 collagen and proteoglycans. The proteoglycans consist of a protein backbone onto which are attached glycosaminoglycans. The most common glycosaminoglycan side chains include chondroitin-6-sulfate and keratan sulfate. These molecules are highly polarized, creating space within the proteoglycan units. The resulting proteoglycans are then aggregated by hyaluronic acid, creating extremely large, highly charged complexes that exert a high osmotic pressure, retaining water within the nucleus (107). Type 2 collagen can interact with carbohydrates, and thus the network of type 2 collagen found within the nucleus pulposus is associated with the glycosaminoglycan side chains, lending stability in the face of compressive forces.

### The Transitional Zone

This zone represents the transition from nucleus pulposus to the annulus fibrosus. Chondrocyte like cells can be seen as well as increasing numbers of fibrocyte like cells moving peripherally away from the nucleus pulposus (22, 108). These cells lie within a fibrous matrix that appears distinct from the more basophilic matrix of the nucleus pulposus. As the transitional zone blends into the annulus fibrosus the fibrous matrix becomes organized into a lamellar orientation.

### The Annulus Fibrosus

The annulus fibrosus consists of inner and outer regions, that are both characterized by concentric fibrocartilage lamellae. These lamellae have elongated fibrocytes interspersed between well-organized bundles of collagen with a less well-organized network of elastin fibers found throughout (22, 108). The collagen in turn has a proteoglycan coating and the healthy annulus fibrosus is 60% water as a result. The regions are differentiated by the presence of chondrocytes in the inner annulus fibrosus and the presence of increasing amounts of type 1 collagen in the outer annulus fibrosus (109). The inner annulus fibrosus is anchored to the cartilaginous endplates and the outer annulus fibrosus anchored to the epiphyseal bone of the adjacent vertebrae by Sharpey's fibers. None of the regions discussed thus far have a blood supply but there is light innervation of the outer annulus fibrosus (9, 104).

### The Cartilaginous Endplates

The cartilaginous endplates firmly anchor the intervertebral disc to the adjacent vertebra and provide additional shock absorbing functionality. The endplates have ~5 layers of chondrocytes and contribute ~6% of the total width of the intervertebral disc (22). They lie immediately adjacent to a rich vascular network from the epiphyseal arterial supply from which nutrients gain access to the IVD. This occurs by osmosis, diffusion and for larger molecules, the central concave regions of the endplates have channels that allow passage of nutrients via bulk flow in response to loading of the disc (22, 104, 110).

## Intervertebral Disc Degeneration

Intervertebral disc degeneration underlies the most common forms of IVD herniation and as such is an extremely important process to understand. Degeneration is effectively an aging process that is heavily influenced by canine genetics and accelerated by biomechanical strain and trauma among other things (9, 110, 111). Overall the process involves replacement of notochordal cells within the nucleus pulposus by chondrocytes with transformation to fibrocartilage (chondroid metaplasia) (5, 14). This is associated with loss of proteoglycans, in particular chondroitin sulfate, and dehydration (12, 107, 111, 112). The resulting biomechanical failure of the intervertebral disc unit is associated with fissuring of the annulus fibrosus and sclerosis of the endplates (5, 12, 110). Throughout this process, the collagen content increases and more type 1 collagen is found toward the center of the disc (Supplementary Figure 1) (8, 12, 111). Complete failure of this unit occurs with IVD herniation.

## Types of Intervertebral Disc Herniation

To be consistent with the majority of veterinary and human literature, we are using the term IVD herniation here to describe any form of IVDD that involves the localized displacement of part of the IVD, typically into the vertebral canal (Table 1). The following are summaries of the key pathological, clinical and diagnostic features that can be used to discriminate between these types of IVD herniation.

### (Hansen Type I/Acute) Intervertebral Disc Extrusion

Historically most often referred to as Hansen Type I IVD disease, herniation or extrusion, this is the most common cause of spinal cord injury in dogs (113, 114). Recently this type of herniation has widely been referred to simply as IVD extrusion, often with the prefix of “acute” applied to indicate the typical clinical presentation and to discriminate from more chronic manifestations of IVD extrusion. The term “extrusion” in this context is defined in Table 1. As a result, in this report we have used this term to describe cases with IVD herniation due to chondroid metaplasia and calcification of the disc, with other reported terms listed for reference in Table 2.

#### Pathophysiology

Early studies into canine IVDD described a characteristic chondroid degeneration of the IVD that was particularly prevalent in certain dog breeds, such as French Bulldogs, Dachshunds and Pekingese (5). These dog breeds all have features of altered endochondral ossification with shortened long bones and along with others such as Beagles, Basset Hounds, Cocker Spaniels, and Pembroke Welsh Corgis, have become known as chondrodystrophic breeds. The degenerative change seen in the IVD is characterized by an early onset of progressive dehydration and calcification, with the normally hydrated and notochordal cell-rich gelatinous nucleus pulposus transforming to a dense, dehydrated cartilaginous matrix rich in chondrocyte-like cells by 1 year of age (5, 9). As an extension of this process, the nucleus pulposus becomes calcified and can be identified clearly on spinal radiographs. This is thought to represent dystrophic calcification of necrotic tissue and occurs predominantly but not exclusively in chondrodystrophic breeds of dog. In breeds such as the dachshund, the number of IVDs with calcified nuclei peaks between 24 and 27 months of age and then decreases (115–117). This chondroid metaplasia and calcification ultimately results in a type of IVD herniation whereby the calcified nucleus pulposus acutely extrudes through a ruptured annulus fibrosus into the vertebral canal (Supplementary Figure 2) (5). This eventual sudden extrusion of degenerative nucleus pulposus into the vertebral canal is a consequence of focal degenerative changes within the annulus fibrosus, hypothesized to be the result of altered biomechanics of the intervertebral disc unit, resulting in separation of the lamellae within the (particularly dorsal) annulus (5, 9, 12). The presence of calcified nucleus pulposus on radiographs is now well-established as an indication that chondroid IVD degeneration has occurred and that there is an increased risk of acute IVD extrusion at that site (115). While overwhelmingly recognized in chondrodystrophic breeds, it is important to note that calcified disks can occur in large breeds, frequently at a single site (5, 118). However, it is unclear whether such disks underwent the extremely early chondroid metaplasia seen in chondrodystrophic breeds (62).

Several advances have since been made in the understanding of the pathophysiology of IVD extrusion, explaining the dramatic early chondroid metaplasia and calcification that occurs in chondrodystrophic breeds. The most important discovery in recent years has been the identification first of a locus on chromosome 12 associated with disc calcification in Dachshunds (119), and subsequently the identification of an expressed *FGF4* retrogene at that locus associated with IVD extrusion in chondrodystrophic dogs (16, 17). Breeds with the most extreme short-limbed body conformation, such as Dachshunds, also carry an *FGF4* retrogene insertion on chromosome 18 that is associated with chondrodysplasia, the condition that produces extremely short limbs (15). The previously documented association between IVD extrusion and body conformation in Dachshunds (120), likely reflects both the influence of the *FGF4* retrogene and the importance of biomechanics in these breeds. It has also been suggested that lifestyle factors such as moderate intensity exercise and stair climbing may be associated with a reduced rate of IVD calcification in Dachshunds (121). The genetic investigations and discoveries related to canine IVDD are covered in more detail in the article in this series by Dickinson and Bannasch. These findings explain the extremely early chondroid metaplasia that occurs in chondrodystrophic breeds, but do not explain the single calcified disks that can be identified in large breed dogs (14, 15, 62, 118, 122).

When using the term Hansen Type I IVD extrusion, we are referring to an acute extrusion of degenerative nucleus pulposus, with features of dehydration and cartilaginous calcification, whilst acknowledging that our current understanding of the underlying pathophysiology might be incomplete. Although acute herniations of material from an IVD that has undergone predominantly fibroid degeneration can occur, this is much less common. When the etiology is unclear, the term IVD herniation can be used without specifying the known or presumed underlying pathophysiology (Table 1).

#### Clinical Presentation and Diagnosis

Young to middle-aged chondrodystrophic dog breeds are most commonly affected by IVD extrusion, although as outlined above, they can also occur in non-chondrodystrophic dog breeds (62, 118, 122). Whilst much less frequently reported and less well-described, IVD extrusions with similar features have also been reported in cats (82, 123). The extruded material causes a variable degree of spinal cord contusion and compression, as well as compression of nerve roots and inflammation (124). An extrusion can occur anywhere along the vertebral column, with an increased incidence of IVD extrusion between the T11-12 and L2-3 IVDs (41). Clinical signs reflect the location of the extrusion along the vertebral canal and can range from mild discomfort with no neurological deficits to paralysis of the affected limbs with loss of pain perception. The typical clinical presentation is therefore an acute onset, painful and progressive myelopathy.

Diagnosis of IVD extrusion has evolved markedly over time and is now mostly commonly achieved using either computed tomography (CT) or MRI (Supplementary Figure 2). Both imaging modalities have been shown to be superior to techniques such as plain radiographs and myelography (Supplementary Figure 2) in diagnosing and localizing IVD extrusions (35, 37, 125). Whilst both MRI and CT can be used to diagnose IVD extrusion, MRI has the benefit of allowing evaluation of soft tissues such as the spinal cord and intervertebral disks (39, 126). The reader is directed to the paper by Da Costa and others in this series for further details on diagnostic imaging in IVDD.

The following is a sub-classification of IVD extrusion, where similar pathological changes of chondroid metaplasia and calcification are likely to underpin the extrusion but result in a different clinical presentation.

### Acute Intervertebral Disc Extrusion With Extensive Epidural Hemorrhage

Acute thoracolumbar IVD extrusions can cause multilevel epidural hemorrhage due to laceration of the internal vertebral venous plexus (37, 67, 127, 128). Sometimes this hemorrhage can be dramatic and can cause multilevel spinal cord compression, and indeed appear as a hematoma (67, 68, 127, 128). The term disc extrusion with extensive epidural hemorrhage (DEEH) has been coined to describe this particular phenomenon (67).

#### Pathophysiology

The affected intervertebral disc undergoes chondroid degenerative changes and calcified nuclear material is extruded into the vertebral canal causing a laceration of the internal vertebral venous plexus and consequent hemorrhage. This phenomenon has been reported in the thoracolumbar spine but not in the cervical spine. The factors that cause ongoing hemorrhage to occur in affected dogs have not been well-defined but likely relate to the relative volume of epidural space. This event is more common in medium to large breed dogs than small chondrodystrophic breeds, leading to speculation that epidural volume is larger in these breeds and thus the vertebral venous plexus is not compressed enough by the extruded material to stop hemorrhage when lacerated. Another possibility is that there is a relatively larger volume of calcified material herniated in chondrodystrophic breeds, effectively compressing the venous plexus to halt hemorrhage.

#### Clinical Presentation and Diagnosis

Typically, affected dogs develop acute paraparesis that rapidly progresses to paraplegia, often associated with severe spinal pain (67). Medium to large sized breeds of dog such as the pit bull terrier, American Staffordshire terrier, Labrador retriever, German Shepherd dog and Rottweiler are affected most commonly, but it can occur in small breed and giant breed dogs as well (52, 67, 68, 128). Survey spinal radiographs might reveal calcified nuclear material and narrowing of an intervertebral disc space. Diagnosis is by advanced imaging with classic findings on MRI including evidence of calcified disc material fragments (T2 and T1-weighted hypointense) and multilevel extradural compression by a mass that is hyperintense or has mixed intensity on T2-weighted imaging and is hyper, iso or hypointense on T1-weighted imaging with variable degrees of peripheral contrast enhancement. Gradient echo (T2^*^) imaging confirms the presence of hemorrhage. The mass often appears like a worm extending along the spinal cord on the sagittal T2-weighted image and gives the appearance of a second spinal cord lying alongside or draped over and compressing the real spinal cord on transverse images. On CT imaging the hemorrhage/hematoma is identified as a moderately hyperattenuating [70–90 Hounsfield units [HU]] extradural mass extending over multiple vertebral levels and lying dorsal, ventral and lateral to the spinal cord. Fragments of calcified disc material identified by higher HU (>100) can be found within the extradural mass, frequently focused over an intervertebral disc space (Supplementary Figure 3).

### (Hansen Type II/Chronic) Intervertebral Disc Protrusion

Traditionally this type of IVD herniation has been referred to as either Hansen Type II IVD disease, herniation or protrusion (Table 2). However, in recent veterinary literature it has been increasingly referred to as simply IVD protrusion, which is the terminology we have used here. The key pathological and clinical features that characterize this type of IVD herniation are outlined below, as well as the areas requiring further investigation.

#### Pathophysiology

In his investigations into IVDD in dogs in the 1950s, Hansen found that dogs could be grouped according to the type of disc degeneration seen most frequently, into chondrodystrophic and non-chondrodystrophic dog breeds (5). In non-chondrodystrophic dogs, with increasing age, Hansen reported that the disc underwent a slow maturation, whereby the collagen content increased and notochordal cells became more fibrocyte-like, a process termed fibroid metaplasia (5). This fibroid metaplasia typically occurred in non-chondrodystrophic dog breeds over 7 years of age, suggesting that this type of IVD degeneration represents a consequence of later onset age-related changes in comparison to chondrodystrophic dogs (5, 110). At the same time as the nuclear degeneration, it has been postulated that small separations develop in the lamellae of the annulus fibrosus, potentially exacerbated by repeated minor trauma, allowing this degenerative fibroid nuclear material to extend into and between the fibers of the annulus (5, 9, 110). The result was a gradual, discrete thickening and protrusion of the surface of the annulus fibrosus, typically occurring dorsally into the vertebral canal, displacing the dorsal longitudinal ligament and slowly compressing the spinal cord (Supplementary Figure 4) (5, 9, 22, 110).

Based on Hansen's description, this type of herniation of the IVD into the vertebral canal became widely referred to as Hansen Type II IVD disease or herniation. However, the distinction between the degenerative processes leading to IVD herniation in the IVD of chondrodystrophic (Hansen Type I) and non-chondrodystrophic (Hansen Type II) dog breeds has been revisited recently. Histopathological comparisons between the IVD of chondrodystrophic and non-chondrodystrophic dog breeds have found features of chondroid metaplasia (chondrification and replacement of notochordal cells by chondrocytes within the nucleus pulposus) in both groups of dogs (13, 14). Investigators have been careful to point out that Hansen's original descriptions had referred to overall degree of fibrosis of the disc, not fibroid metaplasia of the nucleus pulposus specifically, which perhaps had been somewhat misunderstood for many years (14).

IVD protrusion is also often seen in association with other degenerative changes in certain complex disorders of the canine vertebral column, such as disc-associated cervical spondylomyelopathy and degenerative lumbosacral stenosis (71, 129). Whilst a complete discussion of these disorders is beyond the scope of this series, it is likely that nuclear degeneration and annular protrusion occurs as part of a multifactorial etiology in these dogs.

#### Clinical Presentation and Diagnosis

The clinical presentation of dogs with IVD protrusion is dependent on the location of the affected IVD and the degree of associated compression of relevant structures, such as the spinal cord and nerve roots. Clinical signs tend to reflect the chronic, slowly progressive nature of the IVD degeneration, typically with milder neurological deficits than those seen with acute spinal cord injury secondary to IVD extrusions (63). The characteristic clinical picture is therefore that of a slowly progressive, often non-painful myelopathy in an older, usually non-chondrodystrophic dog (63, 64). Pain can be present depending on the presence of nerve root compression but is less common than in the more acute IVD extrusion.

Changes associated with IVD protrusion on plain radiographs include non-specific signs of IVD degeneration such as vertebral endplate sclerosis, spondylosis deformans and IVD space narrowing (63). Although historically diagnosed using myelography, this has largely been superseded by advanced imaging techniques, particularly MRI in the case of IVD protrusion (Supplementary Figure 4) (23, 130). Whilst a specific diagnosis of IVD protrusion can be challenging, MRI criteria have also been reported to assist in differentiating between IVD protrusion and IVD extrusion in dogs (23, 51). Recent studies have also demonstrated the utility of MRI in grading the degree of IVD degeneration using a validated MRI grading scheme, however the grade of degenerative changes seen in IVD protrusions and extrusions were similar (13, 131). Further details regarding diagnostic imaging in IVD protrusions can be found in the paper by Da Costa and others in this series.

### Hydrated Nucleus Pulposus Extrusion (HNPE)

A relatively recent addition to the classification of disorders of the IVD in the veterinary literature has been the description of acute compressive HNPE in dogs (20). This term refers to a subtype of acute herniation of a volume of partially or non-degenerate nucleus pulposus that results in a varying degree of extradural spinal cord compression (20). Whilst the condition has been referred to as “acute compressive HNPE” to signify this compression (84), it is typically referred to by the shortened initialism of HNPE (Table 2). As with ANNPE, there has been variation in the terminology used to describe this presentation, with initial reports of dogs with similar clinical and diagnostic imaging findings using terms “intraspinal cyst” and “canine discal cyst” (86, 87, 132). There has also been a suggestion to abandon either these terms or HNPE in favor of “partially degenerated disc extrusions” on the basis of histological and cytological findings (85), but most recent literature is consistent in using the terminology of HNPE for this condition (Table 2) (81, 84, 133, 134).

#### Pathophysiology

Reports of acute compressive HNPE in dogs typically describe the presence of well-hydrated extradural material overlying an IVD, suggesting a communication with the annulus fibrosus, as well as associated spinal cord compression (20, 86). The previous use of terms such as intraspinal or discal cysts in dogs has its origin in the observation of MRI similarities with human intraspinal discal cysts (86). People with discal cysts most often present with characteristic clinical signs of a chronic and painful radiculopathy, often affecting the lumbar region (135). Human discal cysts are also typically associated with histological evidence of a well-defined cyst wall and the contents are of a serous or serosanguinous nature (135). In contrast, histological and cytological examination of the extradural material in dogs reveals partially degenerated nucleus pulposus (Supplementary Figure 5), whilst a convincing cyst wall has not been consistently identified (81, 83, 133). Furthermore, diagnostic imaging features and microsurgical findings in dogs with HNPE suggest that the extradural material may persist ventral to, or within the dorsal longitudinal ligament (20, 81). Given that dogs with presumed HNPE present with a more acute onset of typically non-painful clinical signs, in combination these findings support the suggestion that the underlying pathophysiology in these dogs represents an acute herniation of hydrated nucleus pulposus (20, 81, 134). However, the extradural material can demonstrate a varying degree of fluidity, detectable on MRI using FLAIR or HASTE sequences, and a good explanation of this range of findings has not yet been forthcoming (Supplementary Figure 5).

The exact mechanisms that lead to this herniation have not been established, but there may be similarities with ANNPE whereby a small tear in the annulus fibrosus occurs following acute changes in intradiscal pressure (81, 133). The differences in clinical presentation of HNPE compared to ANNPE, as well as the reported predisposition for the cervical region suggest that this understanding of the underlying pathophysiology is incomplete and worthy of further investigation.

#### Clinical Presentation and Diagnosis

The vast majority of reported cases of canine HNPE in the veterinary literature have occurred in the cervical vertebral column, suggesting an anatomical predisposition reflected in the typical clinical signs (19, 20, 83, 136). Dogs therefore most often present with an acute onset of tetraparesis or tetraplegia, with symmetrical clinical signs more common in contrast to the lateralisation seen with ANNPE (20). Another characteristic finding reported in dogs with HNPE has been a lack of spinal hyperaesthesia in the majority of cases, as well as more severe neurological deficits in contrast to Hansen Type I IVD extrusion (19, 20, 81, 83, 84). Indeed, tetraplegia with respiratory compromise is not unusual in cervical HNPE (20). No specific breed predisposition has been identified, with both chondrodystrophic or non-chondrodystrophic breeds reported, whilst affected dogs are typically middle-aged or older (19, 20, 136). In most cases the acute onset seen in HNPE appears to occur spontaneously, without inciting causes such as intense exercise or trauma, further differentiating the clinical presentation from that of ANNPE.

Whilst the clinical presentation may provide a high index of suspicion for a diagnosis of HNPE, differentials usually include IVD extrusion, ANNPE and fibrocartilaginous embolic myelopathy (FCEM). Advanced imaging can be used to make a diagnosis of HNPE, with MRI the imaging modality of choice (Supplementary Figure 5). Reported characteristic MRI features consistent with HNPE are listed below (20):

- Ventral, midline extradural material (T2-weighted hyperintense, T1-weighted hypointense) overlying an IVD
- Associated spinal cord compression, with or without intramedullary T2-weighted hyperintensity
- Characteristic bi-lobed “seagull” shaped appearance to the extradural material
- Reduced volume of T2-weighted hyperintense nucleus pulposus signal in the affected IVD.

Although most reports of canine HNPE use these MRI features, a recent study has suggested that contrast-enhanced CT can also be used to make a diagnosis of HNPE with a sensitivity of 91% and specificity of 100% (136).

### Acute Non-compressive Nucleus Pulposus Extrusion (ANNPE)

There has been considerable variation in the terminology used historically to describe this condition, including: traumatic IVD extrusion, IVD explosion, traumatic IVD prolapse, missile disks, high-velocity low-volume disc extrusion and (Hansen) Type III IVDD (18, 75–78). In most of these reports, clinical and diagnostic imaging descriptions suggest a peracute onset extrusion of non-degenerated nucleus pulposus leading to spinal cord contusion with minimal compression, usually at exercise, with or without evidence of trauma. As a result, the term ANNPE has become widely accepted in the veterinary literature as the most descriptive terminology for this condition and is therefore used here. Whilst most studies in the veterinary literature involve dogs, ANNPE has also been described in several cats with similar clinical and diagnostic features (75, 80, 137).

#### Pathophysiology

Within the non-degenerate, normal IVD, the nucleus pulposus draws water down a strong osmotic gradient, creating an innately high intradiscal pressure (22). The surrounding annulus fibrosus on the other hand is composed of dense fibrous tissue with a complex lamellar structure, providing structural integrity as well as mobility (22). As a result, when the IVD is subjected to supra-physiological forces such as those exerted on the vertebral column during brief moments of strenuous exertion or blunt trauma, the annulus fibrosus may tear leading to a sudden extrusion of nuclear material (88). It is hypothesized that the hydrated nuclear material impacts the overlying spinal cord with great force, leading to contusive injury before dissipating or being resorbed due to its hydrated nature and small volume, resulting in minimal or no residual spinal cord compression (18). Reported clinical signs and MRI features reflect this, with several studies describing similar characteristic features (Supplementary Figure 6) (18, 69, 70). Although reports of histopathological confirmation of ANNPE are rare, post-mortem findings have revealed small tears in the dorsal annulus fibrosus in affected dogs and non-degenerated nucleus pulposus material within the vertebral canal, supporting this theory (78).

#### Clinical Presentation and Diagnosis

The characteristic clinical presentation of dogs and cats with ANNPE consists of a peracute onset of signs of myelopathy (ranging from paresis to plegia), usually occurring at strenuous exercise or related to external trauma (18, 69, 138). Clinical signs are lateralised in up to 90% of affected dogs, and are usually non-progressive after the first 24 h (18, 69, 77). Although there are fewer descriptions of feline ANNPE in the literature, more cats have been reported to present with symmetrical clinical signs compared to dogs, and up to 75% of cats with ANNPE present following external trauma (137). Whilst owners may report vocalization at the onset of signs in dogs, clinical examination usually reveals only mild to no spinal hyperaesthesia on palpation (18, 69). In accordance with findings in Hansen Type I IVD extrusion, ANNPE occurs most commonly in the region of the thoracolumbar junction, likely reflecting the increased biomechanical forces at the junction between two stable vertebral segments (18, 69). Whilst clinical signs are usually distinct from the typical presentation seen in compressive IVD extrusions or protrusions described above, an important differential diagnosis for ANNPE in dogs and cats with these clinical signs is an ischaemic myelopathy, most likely reflecting FCEM (69, 88). Spinal MRI is usually required to make a presumptive diagnosis and can be used to differentiate cases of ANNPE from FCEM using specific imaging criteria (88, 139). The imaging findings that can be used to make a diagnosis of ANNPE are listed below (18, 70, 139), with an example shown in Supplementary Figure 6:

- Focal intramedullary T2-weighted hyperintensity of the spinal cord
- Spinal cord lesion located overlying an IVD
- Reduced volume of T2-weighted hyperintense nucleus pulposus signal in the affected IVD
- Mild narrowing of the affected IVD in mid-sagittal view
- Small volume extradural material (T2-weighted hyperintense, T1-weighted hypointense) dorsal to the IVD with minimal to no spinal cord compression.

Whilst ANNPE has been reported in association with external trauma in up to 40% of reported cases (18), there are some specific instances of traumatic IVD extrusion that may require separate consideration. As a result, we have proposed an additional classification of traumatic IVD extrusion as a specific subtype of IVD herniation below to reflect these cases that may present with vertebral fractures or luxations, as well as spinal cord compression in some cases.

### Traumatic Intervertebral Disc Extrusion

Traumatic IVD extrusion (among other terms, also traumatic IVD “explosion” or “prolapse”) has been considered synonymous with ANNPE for some authors, but has also been used to describe IVD extrusion secondary to external trauma (76–78). For the purposes of classification in this article we consider traumatic IVD extrusion as a related subcategory of disorders affecting the IVD. The concept of violent trauma (for example, vehicular trauma) causing a sudden rupture of the annulus fibrosus and subsequent extrusion of disc material into the vertebral canal, regardless of degenerative changes, was highlighted in dogs by Hansen in 1952 (5). At that time Hansen described a case in which “a single extreme violence has ruptured a disc that is not remarkably degenerated,” and proposed the term “traumatic disc prolapse” (5).

Although there have been limited descriptions of this clinical presentation since then, one study identified traumatic IVD extrusions in 62% of dogs with spinal trauma, with spinal cord compression seen in 9 (29%) of 31 dogs, without associated fractures or luxations (77). In this study it was suggested that the spinal cord compression, which can further differentiate these cases from the typical characteristics of ANNPE, was related to chondroid degenerative changes of the affected IVD (140). Whilst histopathological findings were not reported to confirm this, histology is typically not available for such cases and MRI features are often supportive. It is important to note that in cases of external trauma, the disc extrusion may or may not be compressive, likely reflecting the underlying pathology in the IVD at the time of trauma in addition to the traumatic event. As a result, consideration of all aspects of the spinal trauma patient, such as signalment (age, chondrodystrophy) and multiple diagnostic imaging modalities may lead to a specific diagnosis of traumatic IVD extrusion (with or without features of IVD degeneration or spinal cord compression) instead of ANNPE in such cases (Supplementary Figure 7).

### Intradural/Intramedullary Intervertebral Disc Extrusion (IIVDE)

Whilst extruded nucleus pulposus material remains in the extradural space in the case of IVD extrusion, HNPE and ANNPE, there have also been reports of nuclear material penetrating the dura mater (72, 88, 89, 91, 93, 101, 141, 142). The extruded material in this scenario can subsequently remain extramedullary but within the intradural space, or enter the spinal cord parenchyma itself, becoming intramedullary. A recent review article used the term intradural/intramedullary IVD extrusion (IIVDE) to collectively describe these cases, including both cases with and without features of nucleus pulposus degeneration in that classification (88).

IIVDE is an uncommon diagnosis with the largest case series in veterinary literature consisting of 8 dogs, estimated to represent 0.5% of the surgical thoracolumbar IVD extrusion caseload in that study (89). Although most of the reported cases have occurred in the region of the thoracolumbar junction, as with ANNPE and Hansen Type I IVD extrusion, IIVDE has also been described in the cervical vertebral column in dogs and the lumbar region in the cat (93, 141, 143).

Similar to many other reports of IIVDE in dogs (90, 92, 142), that case series describes features of chondroid metaplasia on histological examination of surgically excised nucleus pulposus, suggesting an intradural or intramedullary extrusion of degenerative IVD material and subsequent spinal cord compression (89). However, there are also reports of IIVDE without convincing evidence of advanced degeneration on histological examination, as well as a clinical presentation more fitting with ANNPE, suggestive for IIVDE of non- or partially degenerated nucleus pulposus (72, 88, 101). Some of these cases were originally described as tearing, rupture or laceration of the dura, based on myelography and surgical visualization, with no IVD material identified within the vertebral canal (101). All of these dogs presented with a peracute onset of signs during strenuous activity such as running or jumping, or trauma, typically with an improving clinical course (101, 143–146).

As a result of the similarities in presentation of dogs with IIVDE and either Hansen Type I IVD extrusion, traumatic IVD extusion or ANNPE, antemortem diagnosis is dependent on accurate interpretation of diagnostic imaging, or surgical confirmation of intradural or intramedullary disc material. In most initial reports of IIVDE, myelography was shown to demonstrate either the presence of intramedullary contrast material, a “golf-tee” sign suggestive for intradural-extramedullary material or contrast leakage indicating a dural tear at the site of the presumed IVD extrusion (92, 101). More recent reports suggest that particular imaging characteristics on high-field MRI may be suggestive for a diagnosis of IIVDE, including areas of intramedullary hypointensity on T2-weighted, T1-weighted and gradient echo (T2^*^) sequences overlying an IVD with reduced nucleus pulposus volume, and a linear tract running from the associated IVD to the spinal cord parenchyma (Supplementary Figure 8) (88, 91, 93, 141). A recent case series also suggested that CT-myelography was superior to low-field MRI at diagnosing IIVDE, by allowing the accurate detection of a focal filling defect within the subarachnoid space in cases of intradural-extramedullary disc material (89).

Further investigations into outcome following surgical or non-surgical management of these rare cases are necessary, in which case accurate classification according to the presence of degenerative and non-degenerative IVD material may help to determine if subclassifying types of IIVDE is clinically useful.

## Embolic Disease Associated with the Intervertebral Disc

### Fibrocartilaginous Embolic Myelopathy (FCEM)

Fibrocartilaginous embolism to the vasculature of the leptomeninges and spinal cord was first described in 1973 and since that time, has been a topic of speculation and discussion (103). In the first histopathological descriptions of FCEM, the embolized material was examined closely using several histochemical stains leading to its identification as fibrocartilage (96, 103). Embolization of fibrocartilage can affect the arterial and/or the venous sides of the circulation and results in a peracute onset of often dramatically lateralizing paresis or paralysis (98). The condition occurs most commonly in dogs, accounting for 2% of dogs presenting to an emergency clinic for non-ambulatory paraparesis or paraplegia (114), but it also occurs in a range of other species including the cat (95).

#### Pathophysiology

Of the questions raised about this condition, the first, and most pertinent to this paper, is the unconfirmed source of the embolized fibrocartilage. Although our understanding of the pathophysiology is incomplete, there is a broad consensus that it originates from the underlying intervertebral disc (79, 98, 103). In the first description of the condition it was noted that the annulus fibrosus of a lumbar disc lying beneath the affected spinal cord segments was torn by extruded nuclear material and regions of this torn annulus had identical staining characteristics to the emboli. The author concluded that the annulus fibrosus was the source of the fibrocartilage (103). Subsequently, other authors have proposed it is nucleus pulposus based on histopathology and histochemistry (147–150). However, the normal appearance of the vertebral column on radiography and the intervertebral disks on gross pathology led some authors to conclude that they were unsure of the source (96). More recently, with the widespread use of MRI to image the vertebral column and spinal cord, it has become clear that there are subtle changes in the nucleus pulposus of an IVD close to (frequently just caudal to) the site of the resulting ischaemic myelopathy, lending support to the nucleus as the source of the disc material (Supplementary Figure 9) (95, 139). Indeed, in one study evaluating the use of MRI findings to differentiate between ANNPE and ischaemic myelopathy, a reduced volume of nucleus pulposus in an adjacent IVD was reported in 23.6% of cases with an agreed diagnosis of ischaemic myelopathy (such as FCEM) (139). In addition, the similarity of clinical presentations of medium sized breeds of dog with exercise associated ANNPE and FCEM suggests that peracute extrusion/embolization of nucleus pulposus underlies both conditions.

The second question relates to how the fibrocartilage travels from the intervertebral disc to the leptomeningeal and spinal cord vasculature. In humans a proposed route is herniation of nucleus pulposus into the endplate (Schmorl's nodes) whereby it gains access into the sinusoidal venous channels within the cancellous bone and thus into the basivertebral veins and the spinal cord venous system (151). However, Schmorl's nodes are rare in dogs due to the density of their endplates and have not been described in conjunction with FCEM. Most authors propose extrusion of nucleus pulposus through either healthy annulus fibrosus directly into the internal vertebral venous plexus or spinal arterial vasculature (148, 150), via new blood vessels forming in annulus fibrosus undergoing age related degenerative changes (150), or both (98). The association between FCEM and particular types of exercise that involve jumping and twisting supports the theory that extreme changes in intra-thoracic or abdominal pressure, and therefore intradiscal pressure, result in extrusion of nuclear material directly into the venous or arterial circulation of the spinal cord. Also pertinent to these theories is the marked paucity of reports of FCEM in chondrodystrophic breeds of dog (152–154). It has been hypothesized that the early chondroid metaplasia and ensuing degeneration and calcification of the nucleus pulposus with splitting of the annulus fibrosus results in ready extrusion of nuclear material into the vertebral canal, rather than creating the forces necessary to propel the material into the vasculature.

However, there are numerous reports of cases in which there is no evidence of degenerative changes within the intervertebral disks (96, 98, 103, 155), and exercise or trauma is only reported as an inciting cause in 30% of cases (79). In particular, these theories are less plausible in Irish wolfhounds that develop FCEM as young as 2 months of age, in which it has been proposed that a mismatch between rapidly increasing body weight and the immature IVD results in embolization into the richer arterial supply to the annulus fibrosus of the growing puppy (155). Other unsubstantiated theories for such cases include persistence of embryonal vasculature within the annulus fibrosus and presence of anomalous vessels or arteriovenous fistulae down which fibrocartilage can be embolized (95).

Important histopathological features of FCEM include the presence of numerous embolized vessels, frequently within the leptomeninges, suggesting a shower of embolic material (96, 98, 103, 147, 148). Indeed, the canine spinal cord is resistant to occlusion of single vessels due to its interconnected segmental arrangement and it has been suggested that FCEM will only result when multiple vessels are occluded simultaneously (96). The resulting lesion is one of hemorrhagic ischemic necrosis of the spinal cord that tends to focus on the gray matter, frequently asymmetrically, reflecting the location of the emboli (96, 103).

#### Clinical Presentation and Diagnosis

The typical clinical presentation is that of a peracute onset of non-painful lateralizing signs in non-chondrodystrophic breeds. In the majority of cases signs do not progress beyond 24 h (79). Signs can be cervical or thoracolumbar, and age of onset ranges from 8 weeks to 14 years with males slightly more likely to be affected than females (79). Perhaps the most familiar presentation is the peracute onset of signs during vigorous exercise in medium to large sized breeds of adult (4–6 year) dog such as the Labrador retriever and the Staffordshire bull terrier (69, 95, 99). Indeed, these dogs account for approximately half of all cases (79), and in some case series as many as 80% of dogs (99). The signalment and history of these dogs shows considerable overlap with dogs suffering from ANNPE, with distinguishing features including a slight breed predisposition (e.g., Border Collies more predisposed to ANNPE and Staffordshire Bull Terriers to FCEM) (69). In addition, dogs with ANNPE are more likely to vocalize at onset, to have spinal hyperesthesia on examination, and are more likely to have cervical lesions (69).

There are numerous reports of FCEM in adult giant breed dogs such as the Great Dane (147). Of particular interest is an apparent predisposition in Irish wolfhound puppies (155). Known as Puppy Paralysis or Drag Leg Syndrome within the breed, dogs present between 2 and 4 months of age with a lateralizing cervical or thoracolumbar myelopathy. In some of these young dogs, onset appears to be associated with exercise or minor trauma. Histopathology confirmed fibrocartilaginous embolisation of leptomeningeal vessels (155, 156).

Small breed dogs are also of interest, accounting for 24% of all cases (79). Within these small breeds the Miniature Schnauzer accounts for nearly 60% of all cases with male dogs more at risk than female (94). An association with exercise is less clear in this breed and signs can be cervical or thoracolumbar.

Clinical suspicion is raised whenever a dog presents with a peracute onset of non-painful, lateralizing myelopathy. Antemortem diagnosis is confirmed through MRI of the spine (Supplementary Figure 9) (100, 139, 157). Diagnostic criteria include:

- Focal intramedullary T2 hyperintensity, focused on the gray matter and frequently lateralized.
- Spinal cord lesion overlying vertebral body, not IVD.
- No evidence of extradural material in the region of the lesion.
- Subtle reduction in volume of T2-weighted hyperintense nucleus pulposus signal in the disc caudal to the spinal cord lesion.

CSF analysis can be normal in ~50% of cases, with variable and non-specific changes in the remaining cases. It should be noted that the presence of fibrocartilage within the vasculature can only be identified post-mortem, so the imaging diagnosis is one of ischemic myelopathy. However, based on histopathology, the most common cause of ischemic myelopathy in dogs is FCEM.

## Discussion

Uniform disease definitions and systems of classification are vital tools in order to accurately diagnose patients, as well as to consistently document and report conditions in such a way that allows reliable scientific comparisons and future critique of related research. It is likely that the advancement of techniques such as genetic technology ultimately hold the key to more accurate disease categorization, by completing the path from genotype to phenotype (158). In the meantime, given the introduction of several new conditions related to the IVD to the veterinary literature in recent years, we have aimed to evaluate current systems of classification and terminology used to ensure consistent recording of IVDD types.

The rapidly expanding language used surrounding types of IVDD in the veterinary literature suggests that a consensus on terminology and classification is required to cover multiple types of IVDD, including those falling outside of the traditional Hansen Type I and II system. Whilst there has been a gradual advancement in the terminology used in the veterinary literature to describe these additional types of IVDD, there remains great inconsistency. This limits the ability to perform high quality research or to accurately compare clinical data, as clinicians and researchers are restricted by the lack of clear disease definitions. An improved understanding of terminology can also facilitate clearer communication between colleagues and clients when discussing IVDD. Increasingly, efforts have been made to adapt terminology used in the veterinary literature to include other recently described types of IVDD, as well as drawing from the human medical field with an emphasis on appropriate anatomical descriptions (10). In Tables 1, 2 and throughout this report, we have taken a similar approach to using specific and consistent terminology for each condition. We have aimed to provide a point of reference in matters of IVDD classification moving away from the restrictions of the traditional Hansen Type I and II system, as well as providing our interpretation of the meaning associated with terms currently used in the veterinary literature.

Most systems of disease classification in veterinary and human medicine have origins in historical methods used to document population disease and health statistics, and are usually based on pathological systems of categorization (158). As a result, traditional forms of IVDD classification have focused on degenerative IVDD and the differentiation between Hansen Type I and Hansen Type II IVD, using signalment, clinical presentation and histopathological features (5, 12, 159). Although advanced diagnostic imaging techniques such as MRI have added to the ability to characterize IVD degeneration clinically (12, 13, 126, 160, 161), there are limitations to a system of classification based solely on the determination of degrees of IVD degeneration. For example, acute herniations of an IVD that has features of predominantly fibroid degeneration may provide a challenge with this system of classification. Furthermore, considering recent research suggesting that dog breeds traditionally considered non-chondrodystrophic can also demonstrate features of chondroid metaplasia, it is clear that the distinction of IVDD into the traditional Hansen Type I and Type II labels represents an incomplete picture.

**Figure 1:**
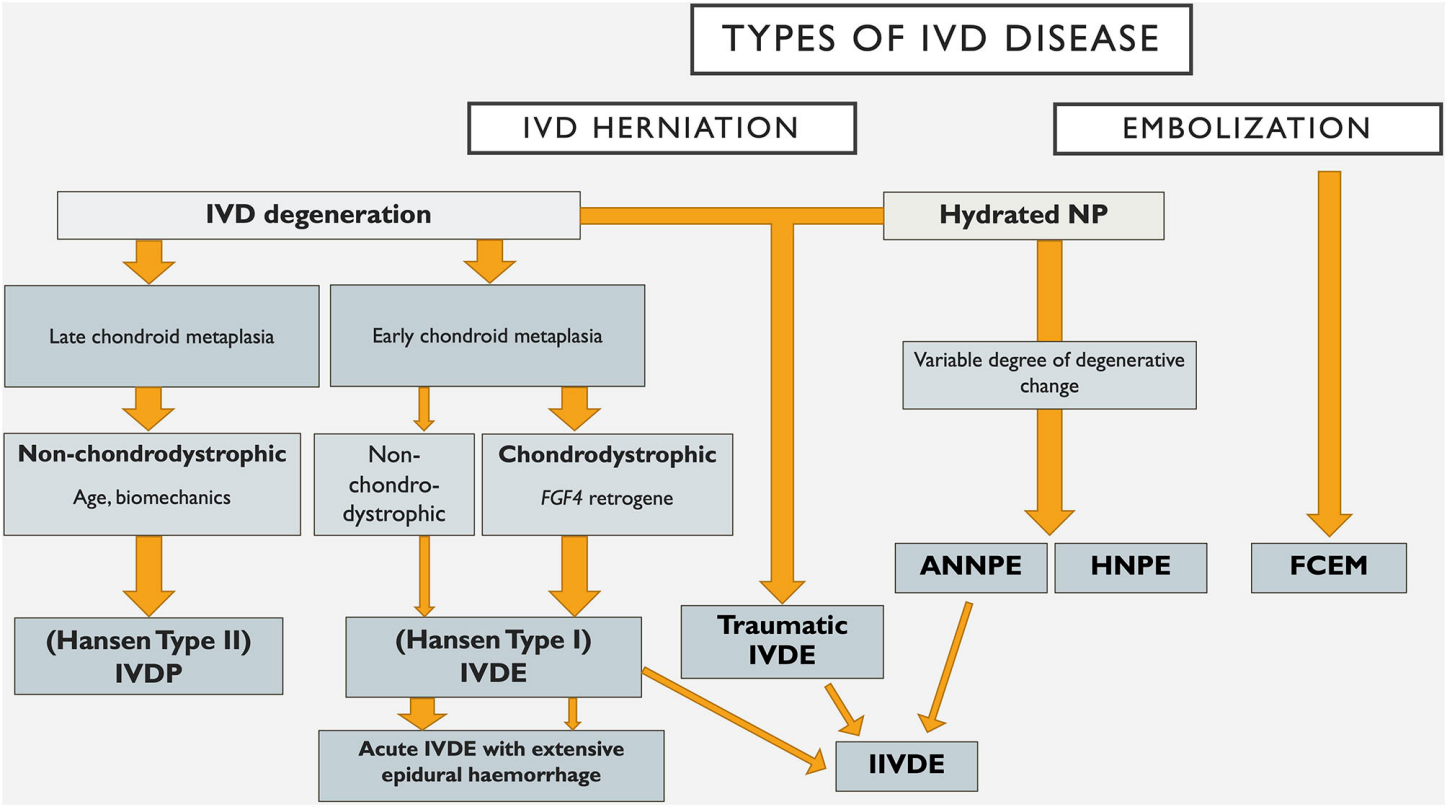
An example of how a classification system for intervertebral disc disease in dogs might evolve. IVD, intervertebral disc; NP, nucleus pulposus; IVDP, intervertebral disc protrusion; IVDE, intervertebral disc extrusion; HNPE, hydrated nucleus pulposus extrusion; ANNPE, acute non-compressive nucleus pulposus extrusion; IIVDE, intradural/intramedullary intervertebral disc extrusion; FCEM, fibrocartilaginous embolic myelopathy.

Within the field of human neurology there are numerous examples of disorders that were classified based on histopathological and clinical findings that have been reclassified based on undercovering the genetic basis of the disease. These include for example, the hereditary ataxias, which take into account the mode of inheritance, the clinical syndrome and the genetic cause (162). Genetic discovery holds similar promise in furthering our understanding of canine IVD degeneration, as evidenced by the identification of the chromosome 12 *FGF4* retrogene as a cause of chondrodystrophy and early, severe chondroid metaplasia with ensuing disc extrusion (15–17). Such advances also enhance our ability to classify IVDD for the purposes of diagnosis, prevention and treatment. An example of how such a system might evolve is provided in Figure 1. It is likely that future IVDD classification systems will utilize further genetic findings, such as *FGF4* genotype, to supersede traditional labeling of certain breeds as chondrodystrophic as a means of subcategorising affected dogs. However, the genetic picture is complex and multifactorial (see Dickinson and Bannasch's paper in this series) and in a degenerative disorder such as IVDD, additional environmental influences clearly play a role. The increasing understanding of the complex processes of IVD degeneration, and somewhat contradictory studies on the degree of degenerative changes occurring with types of IVD herniation such as ANNPE and HNPE makes classification based on degenerative processes less clear (9, 14, 81, 85). In the future we anticipate that advanced genetic, diagnostic imaging and histological investigations will build on recent developments and allow subclassification across a wider range of IVD degeneration.

As an alternative to using pathological features of degeneration, we have explored the possibility of using clinical and diagnostic imaging features to classify primary disorders of the IVD. For example, recent human recommendations on lumbar IVDD nomenclature have proposed using morphological characteristics such as the shape of the IVD herniation to distinguish between IVD extrusion and protrusion, regardless of the underlying pathology (11). Another option would be to categorize IVDD primarily into compressive and non-compressive forms, however these classification systems are also open to degrees of interpretation and require separate categories for conditions with unique aetiologic or anatomic features, such as: traumatic IVDE, intradural/intramedullary IVDE (IIVDE) and embolic disease (FCEM). One advantage of such a system would be the incorporation of terminology that ties in with clinical decision-making. For example, traumatic IVD extrusion may necessitate a specific clinical approach that differs from non-traumatic, compressive IVDD, or embolic disease (FCEM). However, by the same note it is important to consider that the treatment for all conditions with spinal cord compression is not necessarily the same. In such cases treatment is typically based on a combination of factors such as severity of clinical signs, owner preference and financial constraints. More specifically, HNPE is often treated medically despite being compressive, largely based on the assumption that the material is well-hydrated and will therefore dissipate over time without intervention (20, 84). It is also important to consider that given the similarity in clinical presentation between some of these conditions, such as ANNPE and FCEM, future research should aim to achieve a better understanding of the correlation between histological, clinical, and imaging features.

Another possible approach is therefore to classify types of IVDD according to their typical clinical presentation. For example, IVD protrusion would be classified as chronic, non-painful and progressive, whereas ANNPE would be classified as peracute, non-painful, and non-progressive. Whilst combinations of clinical features have been shown to be associated with specific forms of IVDD (163), there are still areas of overlap and variations from the typical clinical findings within several of these categories.

As a result, although an ideal system of classification among types of IVDD does not currently exist, consistency across the veterinary literature in the terminology used for each individual form of IVDD would clearly be beneficial. The authors encourage the consistent use of the terms used in the current series of papers clinically and when describing cases in the veterinary literature. Future research efforts should focus on improving our understanding of the underlying pathophysiology of these different types of IVDD, an endeavor which can be facilitated by a clear understanding of terminology used. It is possible that more advanced systems of characterizing IVD degeneration, in combination with advances in genetic investigations, clinical understanding and diagnostic imaging techniques, will allow a more accurate and uniform system of classifying disease of the IVD in the future.

## Author Contributions

JF and NJO both contributed equally to the design and writing of the manuscript and all CANSORT-SCI members critiqued and approved the final version of the manuscript. All authors contributed to the article and approved the submitted version.

## Conflict of Interest

The authors declare that the research was conducted in the absence of any commercial or financial relationships that could be construed as a potential conflict of interest.
