# Supplementary figures and images for: Classification of Intervertebral Disc Disease
Source: Front Vet Sci. 2020 Oct 6;7:579025. doi: 10.3389/fvets.2020.579025 (PMC7572860; doi:10.3389/fvets.2020.579025)

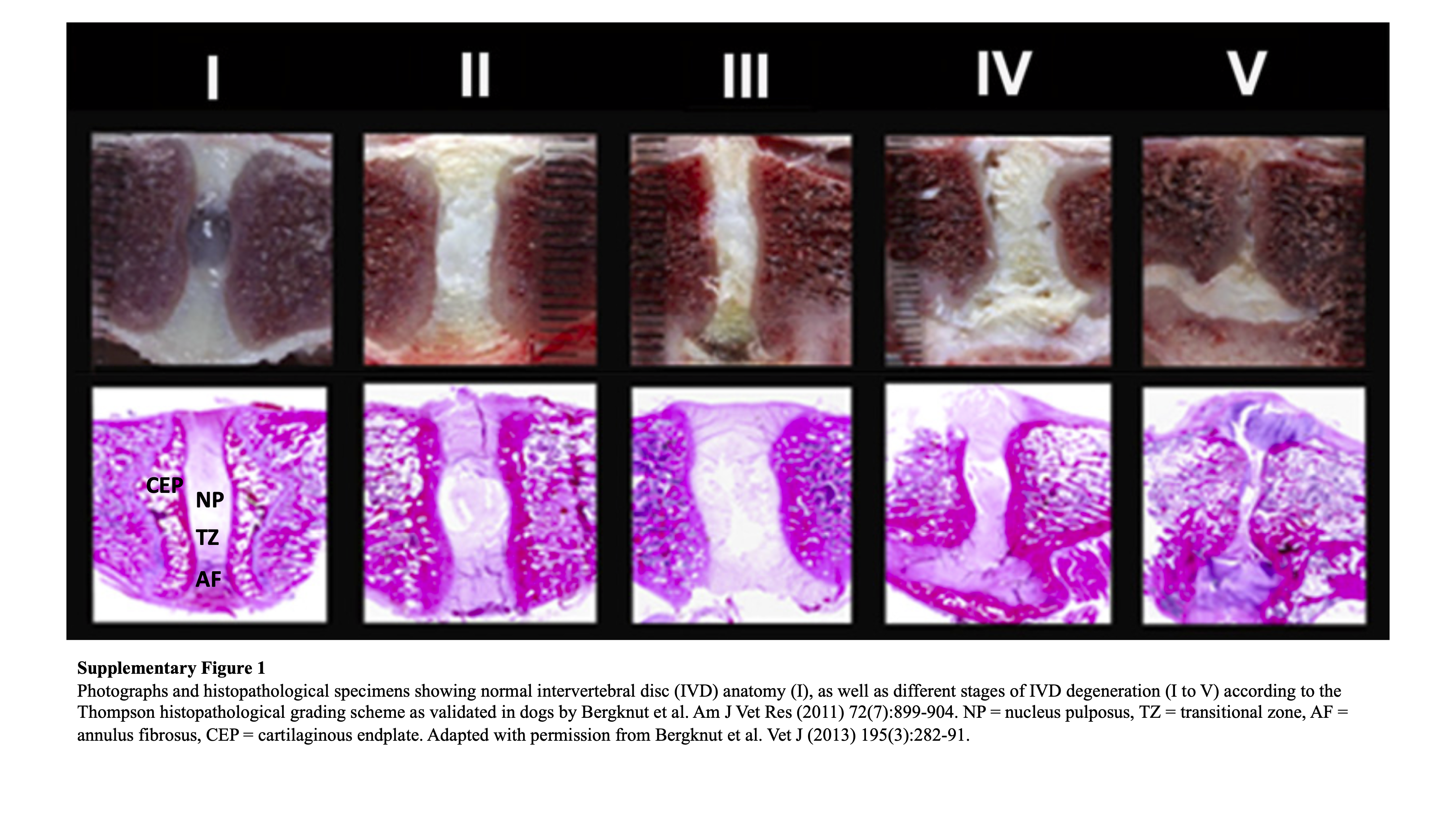

Supplement: Supplementary file 1 [file Image_1.TIFF]

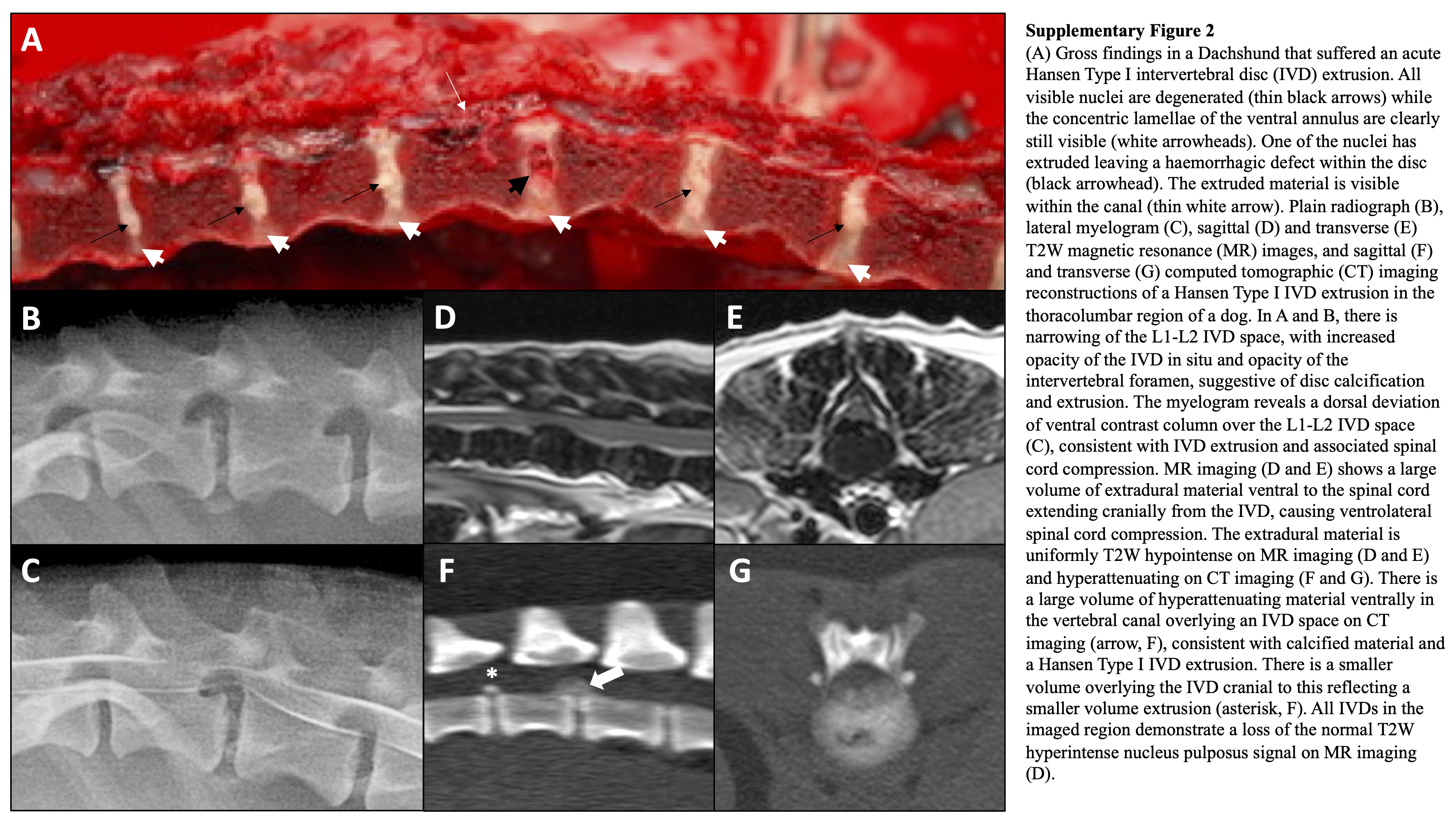

Supplement: Supplementary file 2 [file Image_2.TIFF]

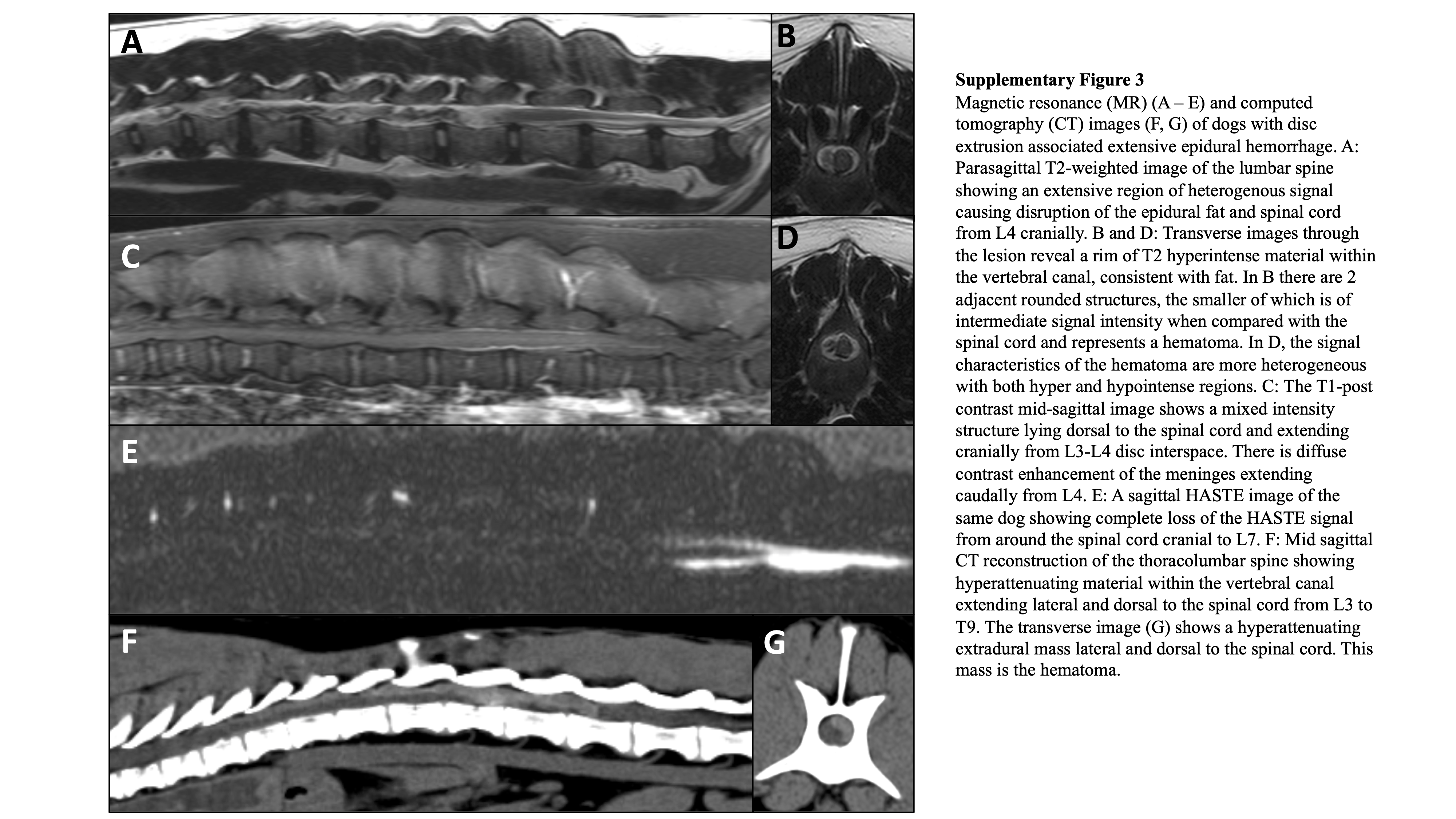

Supplement: Supplementary file 3 [file Image_3.TIFF]

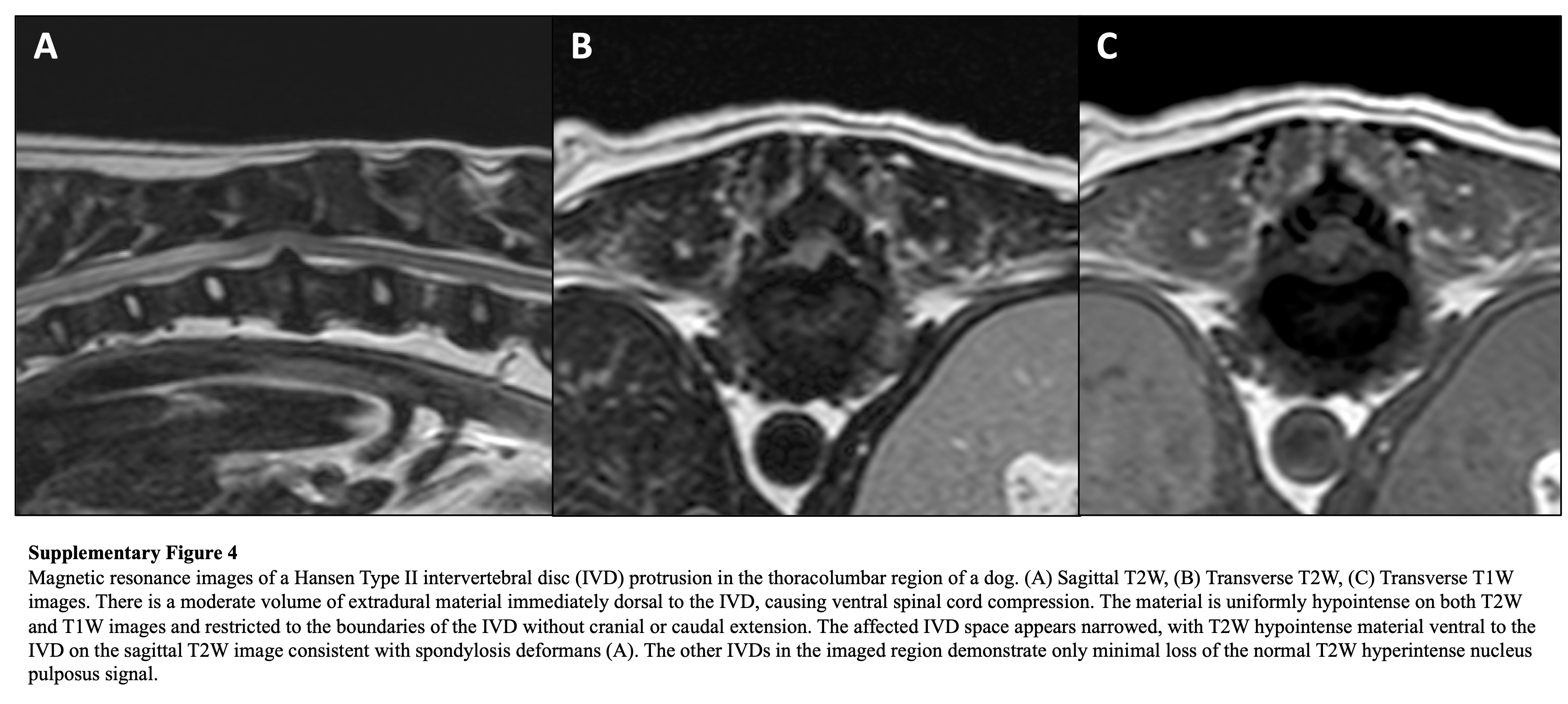

Supplement: Supplementary file 4 [file Image_4.TIFF]

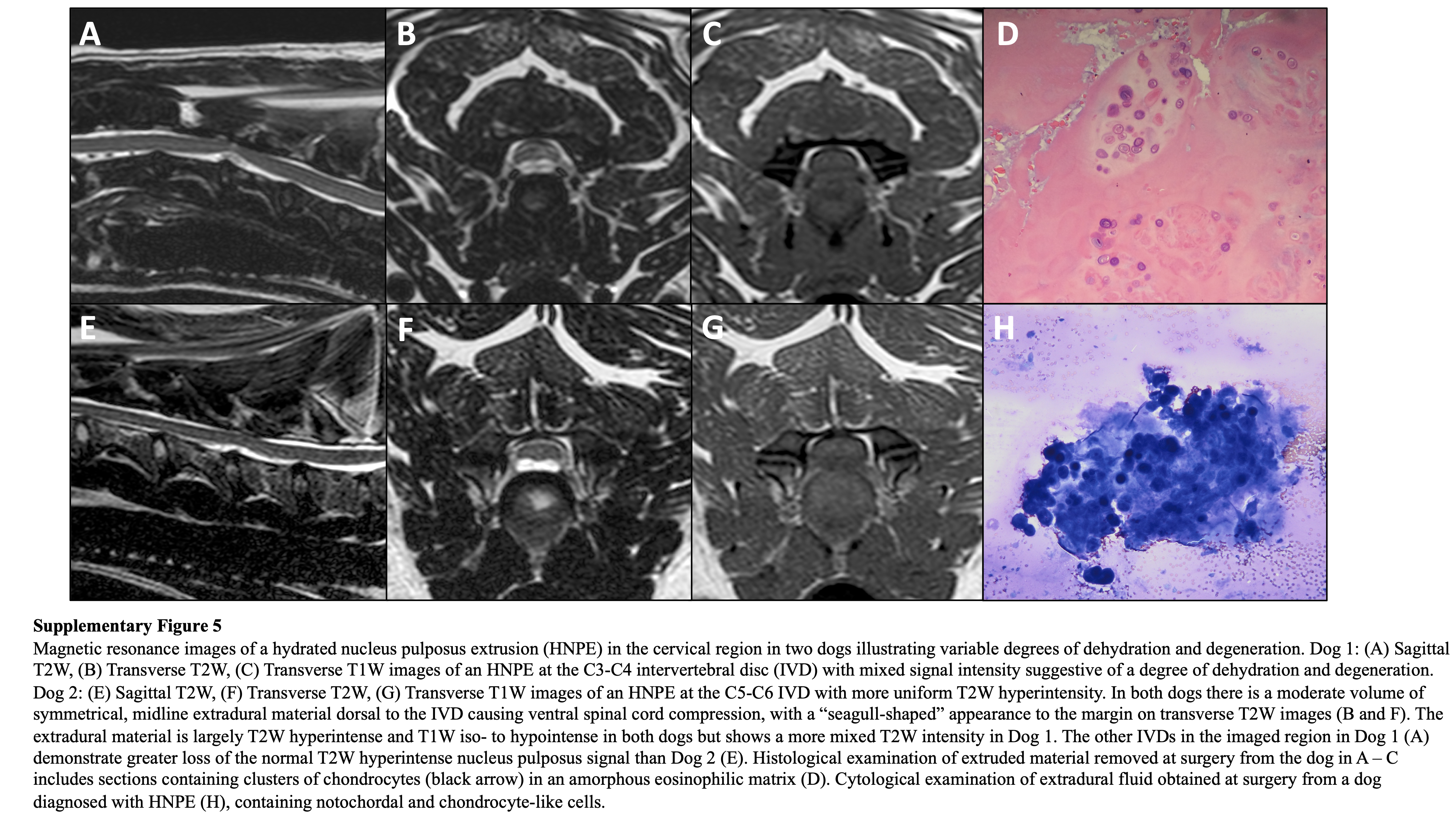

Supplement: Supplementary file 5 [file Image_5.TIFF]

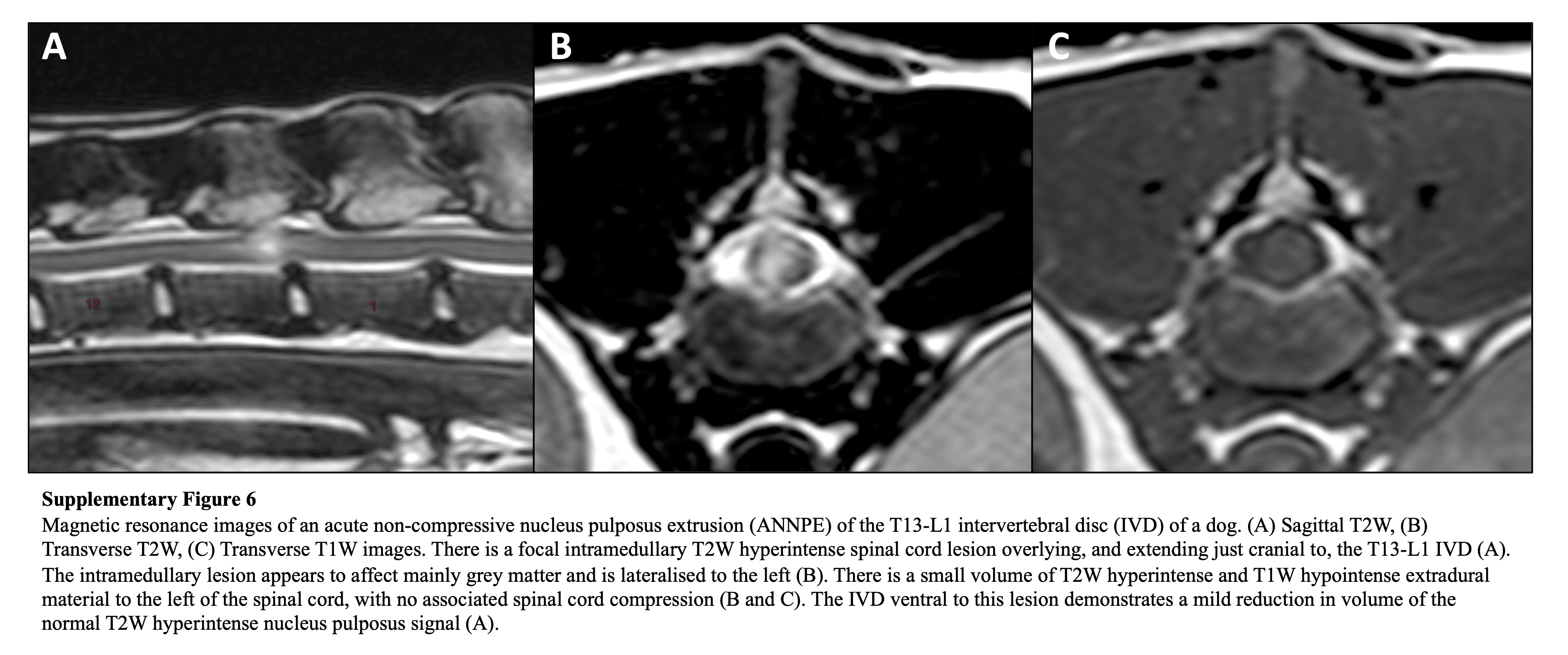

Supplement: Supplementary file 6 [file Image_6.TIFF]

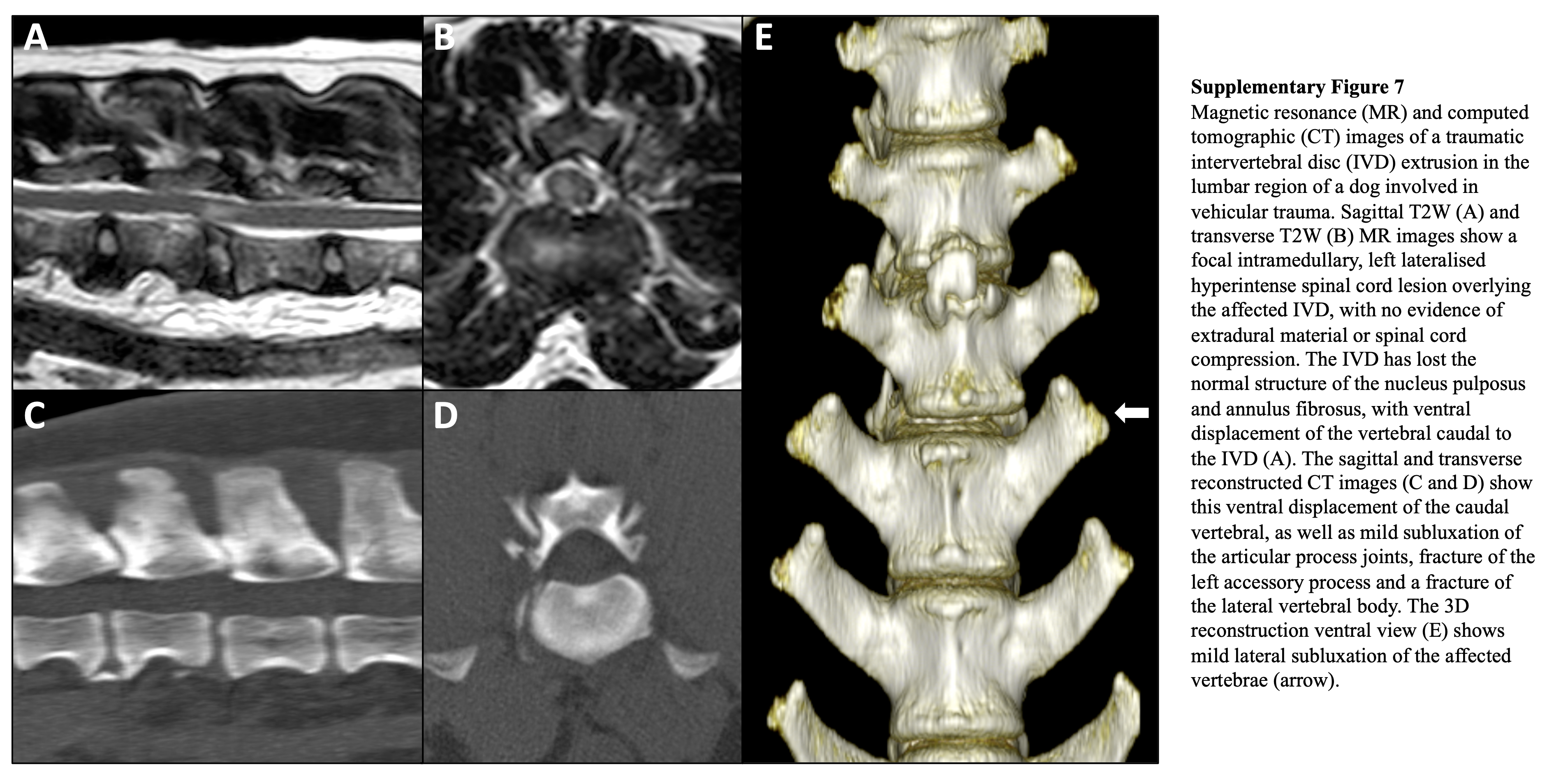

Supplement: Supplementary file 7 [file Image_7.TIFF]

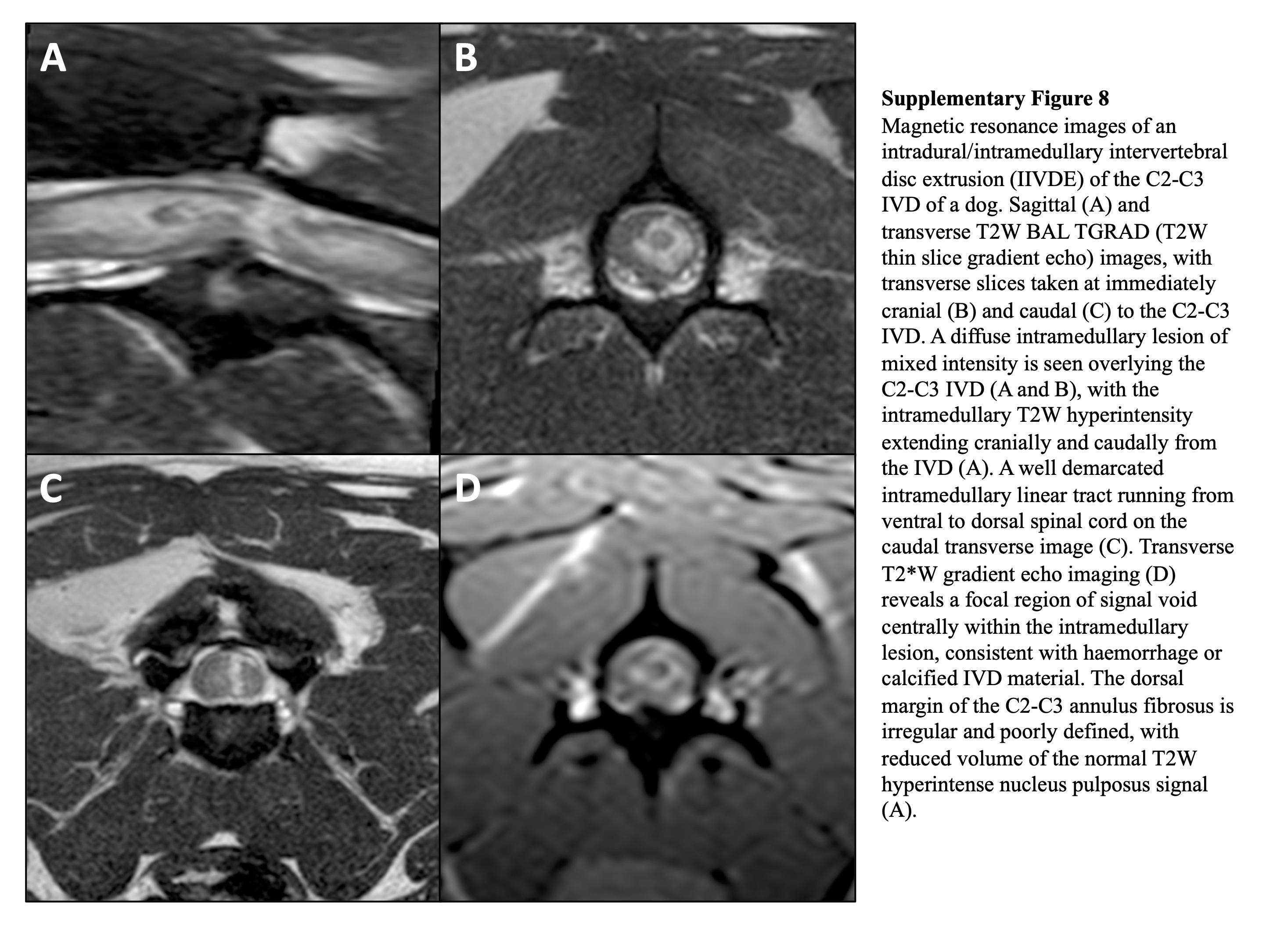

Supplement: Supplementary file 8 [file Image_8.TIFF]

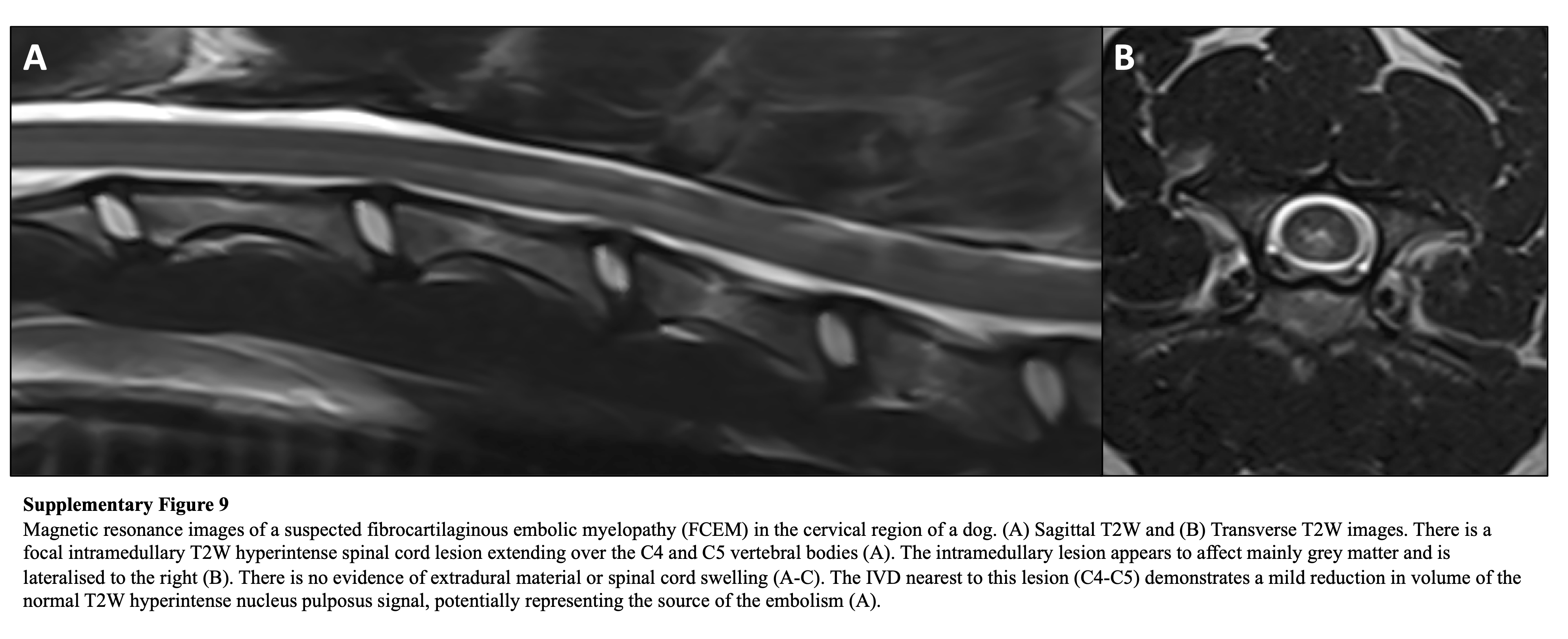

Supplement: Supplementary file 9 [file Image_9.TIFF]
